# The Relationship Between Cognitive Functions and Sport-Specific Motor Skills in Elite Youth Soccer Players

**DOI:** 10.3389/fpsyg.2019.00817

**Published:** 2019-04-25

**Authors:** Hans-Erik Scharfen, Daniel Memmert

**Affiliations:** Institute of Exercise Training and Sport Informatics, German Sport University Cologne, Cologne, Germany

**Keywords:** elite, youth, cognitive functions, soccer, motor skills, sport-specific skills

## Abstract

The aim of the present study was to examine the relationship between basic cognitive functions and sport-specific motor skills in elite youth soccer players. A total of 15 elite youth soccer players aged 11–13 years performed a computer-based test battery measuring the attention window (AW), perceptual load (PL), working memory capacity (WMC), and multiple object tracking (MOT). Another set of tests was used to asses speed abilities and football-specific technical skills (sprint, change of direction, dribbling, ball control, shooting, and juggling). Spearman’s correlation tests showed that the diagonal AW was positively associated with dribbling skills (*r_s_* = 0.656) which indicates that a broader AW could be beneficial for highly demanding motor skills like dribbling. WMC was positively related to dribbling (*r_s_* = 0.562), ball control (*r_s_* = 0.669), and ball juggling (*r_s_* = 0.727). Additionally, the cumulated score of all cognitive tests was positively related to the cumulated motor test score (*r_s_* = 0.614) which supports the interplay of physical and psychological skills. Our findings highlight the need for more, and especially longitudinal, studies to enhance the knowledge of cognition-motor skill relationships for talent identification, talent development, and performance in soccer.

## Introduction

High-demand sports require extraordinary physiological capacities combined with outstanding abilities in the areas of motor control, perception, and cognitive functioning. Two recent meta-analysis (Voss et al., 2010; Scharfen and Memmert, 2019) showed small to middle effects of basic cognitive functions in experts and elite-athletes which may point at their superiority in terms of basal cognitive functions. Besides the physiological abilities, previous research mostly focused on the cognitive skills of elite adult athletes (Mann et al., 2007; Voss et al., 2010; Scharfen and Memmert, 2019). In terms of elite youth athletes, especially soccer players, current research mainly studied – on the one hand – the physical or physiological prerequisites of elite youth soccer players (Unnithan et al., 2012; Waldron and Murphy, 2013; Abade et al., 2014; Murr et al., 2018) or – on the other hand – the psychological prerequisites, that is the cognitive functions of elite youth soccer players (Verburgh et al., 2014, 2016; Balakova et al., 2015; Huijgen et al., 2015; Vestberg et al., 2017) in isolation. To the best of our knowledge, the combination of both motor (i.e., soccer-specific motor skills) and basic psychological (i.e., cognitive functions) has not yet been examined. Therefore, the present study is unique as it connects basic psychological (cognitive functions) with motor (soccer-specific motor skills) aspects of elite youth soccer players.

Cognitive skills refer to the ability to identify and acquire environmental information in order to integrate them with existing knowledge (Marteniuk, 1976). This allows the individual to select and execute the appropriate responses. An especially interesting and important subgroup of these skills are executive functions (EF) which describe cognitive processes that regulate thoughts and actions, especially in non-routine situations (Friedman et al., 2014). The EF are further subdivided into core EF (CEF), which can be defined as working memory, cognitive flexibility and inhibitory control, and higher-level EF (HEF), involving reasoning, problem solving, and planning (Diamond, 2013). These EF skills mature at different ages, as they depend on different prefrontal structures. The neuronal structure underlying HEF is the prefrontal cortex which matures slowly and last in development; full capacity is reached between 20 and 29 years of age (De Luca et al., 2003; Luciana et al., 2005). In contrast CEF develop its total capacity earlier in the lifespan, most often before early adolescence (Crone et al., 2006). We related their motor skills to CEFs [working memory, object tracking, inhibition under perceptual load (PL), and flexibility to widen the attention window (AW)] as these develop earlier than HEFs and may be a key predictor for cognitive functions this early in maturation. Additionally, distinct motor-cognition interactions were proposed with strong mutual influences in terms of (i) functional brain networks (e.g., Leisman et al., 2016; Ptak et al., 2017), (ii) structural brain networks (e.g., Hanakawa, 2011; Koziol et al., 2012; Bigelow and Agrawal, 2015; Gao et al., 2018). More specifically, recent studies show that (1) cognition emerges from motor function in young children (i.e., 1.5–6 years of age) – they predicted several cognitive functions like mental rotation ability (Jansen and Heil, 2010; Lehmann et al., 2014), working memory (Lehmann et al., 2014; Gottwald et al., 2016), inhibition (Gottwald et al., 2016) and (2) that exercise improves cognitive function (Hillman et al., 2009; for review see Tomporowski et al., 2015). Additionally, a review by van der Fels et al. (2015) found weak-to-strong relations between motor and cognitive skills, especially in pre-pubertal children (i.e., under 13 years of age) whereas Hartman et al. (2010) reported correlations between motor performance and EF in children with intellectual disabilities. Grooms and Onate (2016) state that the ability to maintain motor control in the unpredictable sport environment demands a complex central nervous system integration of constantly changing inputs, the processing of which also depends on cognitive functions. However, this study is related to the cognitive skills approach as basic cognitive functions are analyzed. Having the value for talent scouting in mind, the current research sets a focus on youth elite athletes.

However, to the best of our knowledge none of these interactions have been examined on a behavioral level in an elite-sports context yet with children in this development stage (i.e., around 12 years of age). In addition, we are following the call of Leisman et al. (2016) by conducting this examination.

### Review of Literature on Cognitive Functions and Skills in Elite Youth Soccer Players

In the following, several studies regarding cognitive functions within similar study designs are described (i.e., cross-sectional, elite-athletes, youth, and age range from 9 to 14 years). Verburgh et al. (2014), conducted a study with the aim to examine a broad range of cognitive skills in elite and sub-elite soccer players (*n* = 126), with an average age of 11.8 years. They measured cognitive abilities, motor inhibition, alerting and orienting, executive network and executive attention, as well as visuospatial working memory. They used a stop signal task, a modified flanker attention network test, and an adapted Bergman-Nutley task (VTSM forward and backward). They reported heterogeneous results: The elite group outperformed the sub-elite in terms of reaction time in the motor inhibition task as well as in the alerting attention task. No differences were found in orienting attention, executive attention, or working memory capacity (WMC).

In another study, the same research group (Verburgh et al., 2016), carried out a similar investigation of elite and sub-elite soccer players and non-athletes (*n* = 168, *M* = 10.5 years of age); they checked for motor inhibition, verbal short-term memory, working memory, and visuospatial short-term memory. The elite players significantly outperformed the sub-elite players and the non-athletes in terms of inhibition, short-term memory, and partially working memory. Furthermore, the sub-elite players outperformed the non-athletes in terms of short-term memory and working memory. Moreover, Vestberg et al. (2017), investigated 30 elite soccer players (*M =* 14.9) in regard to their cognitive functions. Significant results were found for processing speed, simple attention, and WMC in which the elite players performed highly above the level of the normal population. Additionally, working memory and multiprocessing as well as the combination of both functions positively correlated with scored goals whereas no significant correlation was found between processing speed or attention and scored goals.

In contrast to these findings by Vestberg and colleagues which support superior cognitive functions in youth elite athletes, some other studies did not show this exceptionality. For example, Balakova et al. (2015), studied a wide range of cognitive abilities such as visuospatial short-term working memory, reaction ability, and attention by usage of the Vienna test system in 91 elite soccer players (*M*age = 13). No differences were found between talented and less talented players except for the ability of spatial and temporal movement anticipation. Also, Granacher and Borde (2017), reported no significant expertise differences when testing elite youth athletes (*M*age = 9.5) and non-athletes regarding concentration and attention. For an overview see Table 1.

**Table 1:** Cognitive measurements of elite youth athletes.

Author	Age	Academy/ Country	Type of sport	Test	Measured cognitive abilities	Results
Granacher and Borde, 2017	9,5	Elite-school of sport Germany	Various sports (including soccer)	d2 test	Concentration and attention	*No sign*. Elite differences
Verburgh et al., 2016	10,6	Professional youth soccer academy Netherlands	Soccer	Stop signal task Digit span forward Adapted version of Bergman-Nutley task (VTSM forward)	Motor inhibition Verbal short-term memory (STM) and working memory (WM) Visuospatial short-term memory	Elite *sign*. better than sub-elite and non-athletes: inhibition, STM, WM (not better than sub-elite) Sub-elite athletes *sign*. outperform non-athletes in STM, WM Time spent in organized sports *sign*. positively correlated with inhibition, STM, WM, lapses of attention
Verburgh et al., 2014	11,8	Professional youth soccer academy Netherlands	Soccer	Stop signal task Modified attention network test Modified Flanker task Adapted version of Bergman-Nutley task (VTSM forward and backward)	Motor inhibition Alerting and orienting Executive network and attention Working memory (visuospatial sketchpad and central executive)	Elite *sign*. better than sub-elite (SSRT) but slower RT on go trials Elite with *sign*. larger gain in RT, no differences in orienting attention *No sign*. Elite differences in executive network and attention, working memory
Balakova et al., 2015	13	Professional youth soccer academy Czechia	Soccer	Vienna test system: Reaction test Corsi Block-Tapping test Long-term selective attention test Visual-pursuit test Stroop test Visual memory test Time/movement anticipation test Determination test Gestalt perception test	Cognitive abilities Ability to react Visuospatial short-term working memory Focused attention Visual perception Color-word interference Short-term memory Spatial/temporal movement anticipation Stress tolerance, reactive Special ability test	*No sign*. Elite differences Except the anticipation test (talented within group *sign*. outperformed less talented group)
Vaeyens et al., 2007	14,7	Professional youth soccer academy Belgium	Soccer	Soccer specific video clips (gaze behavior)	Decision making process	Successful within elite group *sign*. quicker in all conditions than less successful group and more accurate in decision making (except condition 2 vs. 1)
Vestberg et al., 2017	14,9	Professional youth soccer academy Sweden	Soccer	CogStateSports Design fluency (DF) Colorword interference test (Stroop test) Trail making test	Demanding working memory (dWM): Attention, processing speed, learning, working memory Multiprocessing (creativity, response inhibition, cognitive flexibility) Cognitive flexibility, verbal inhibition Scanning ability, multiprocessing, cognitive flexibility, short-term memory	Elite players *sign*. above level of normal population: processing speed, attention and dWM No *sign*. Correlation between processing speed / attention and scored goals *Sign*. positive Correlation between dWM, DF, composite score of DF, DWM, and scored goals

### The Present Research

Reviewing the literature on cognitive skills in elite athletes in general, it is conspicuous that there are much fewer studies on youth elite players – especially in the age range from 9 to 14 – (*n* = 6) (see Table 1) than there are on adult elite players – 18 years of age or older – (*n* = 23). This unpublished review was conducted by the authors in February 2018 by using following inclusion criteria: cross-sectional study, elite- or expert-athletes, examination of active athletes, statement of a specific type of sport. Additionally, the reviewed literature on cognitive skills in youth elite athletes reveals conflicting results and heterogeneity in terms of the used tests (a comparison of employed cognitive tests is illustrated in the supplemental material). The present study aims to enrich the literature on cognitive functions in youth elite soccer players by studying the interplay between these functions and motor/technical skills for the first time. Therefore, the purpose of our research is to set a starting point and open new pathways for research and discussion in terms of the link between cognition and motor skills in youth elite athletes. Furthermore, this linkage has been depicted only on brain-structural and functional dimensions. Therefore, it should be analyzed on a behavioral level. More specifically, the aim of this study was to investigate the relation between cognitive functions [working memory, PL, multiple object tracking (MOT), and AW] and soccer specific motor skills (sprint, change of direction, dribbling, ball control, and ball juggling) in soccer players aged between 11 and 13. These cognitive tests are used based on previous research depicting their crucial importance in elite soccer: (1) working memory; (2) PL (e.g., Vestberg et al., 2012;Verburgh et al., 2014, 2016; Huijgen et al., 2015); (3) MOT (e.g., Faubert, 2013; Romeas et al., 2016); and (4) AW (Hüttermann et al., 2014). As this is a first of its kind investigation the sample size is quite small, because this first step in a possible opening of new research pathways should analyze whether it is worthwhile in the first place. If so, future studies would need to examine this in larger populations.

## Materials and Methods

### Participants

A total of 19 elite youth soccer players from the talent development program of the youth academy of a professional German soccer club were recruited. The participants were boys born between 2005 and 2006 (*M*_age_ = 12.72, *SD*_age_ = 0.45) and had started playing soccer at approximately 5.2 years of age (*SD =* 1.4). At the time of data collection, their teams were playing at the top level of their respective age group and the players were part of a professional youth academy for an average of 2.75 years (*SD =* 1.47). Participants were not diagnosed with any behavioral, learning, or medical conditions that might influence cognitive abilities. Four datasets of players had to be excluded, two due to missing motor datasets and two because of their positions as goalkeepers, which highly influenced the motor test in a negative way. Therefore, 15 datasets were used for the study. Written informed consent was obtained from every participant before commencing the experiment. The study was carried out in accordance with the Helsinki Declaration of 1975 and was approved by the ethics committee of the German Sport University Cologne.

### Procedure and Materials

Data of the cognitive tests were collected in a separate and quiet room. The cognitive test session was conducted prior to a soccer training and consisted of one session lasting approximately 1 h with two players performing the tests simultaneously. We used a battery of four tasks to explore individual differences in basic cognitive mechanisms. Each task is described below. The order of the cognitive tests and the different conditions within were randomized. Participants were instructed to sit in a comfortable position leaning against the backrest of the chair, so that the distance to the screen (approximately 45 cm) was the same for all the players. One experimenter tested all players in a standardized process and was blind to the hypotheses. Additionally, the motor performance test was acquired in a gym approximately 4 months prior to the cognitive tests. This difference regarding the time point of measurement exists due to the fact that the motor test was not conducted for the purpose of this study solely as this test battery is part of the German Soccer Association (DFB) talent-development program and is conducted twice a year in every professional youth academy and at the DFB bases of the talent-development program. Therefore, all players did know this test battery already. Test leaders were either licensed soccer coaches of the youth academy or the DFB talent-development program. The data of this motor performance test were used, because they were analyzed and confirmed for objectivity, reliability, and validity in large scale study (N = 68,158) by Höner et al. (2015).

#### Cognitive Tests

For stimulus presentation, E-Prime 1.2 (Psychology Software Tools Inc.) and two 15-inch computer screens with a resolution of 1,024 × 786 pixels were used.

The *attention window task* (AWT) by Hüttermann et al. (2013), was used to assess the individual attention breadth on diagonal axis. During each trial, participants were instructed to fixate a central point [21] and try to spot a white triangle within a circle (1.1° diameter) among square distractors (1.1° × 1.1°). Across trials, the target appeared at varying distances from the fixation point (10°, 20°, and 30°) along one of eight equally spaced radial lines that originated from a square in the center of the display (45° apart). This random display was flashed for 12 ms and was followed by a colorful mask (100 ms). After every mask, subjects were asked to indicate how many white triangles they had just seen in the different locations depending on the orientation of the items. Participants completed 180 trials. This particular task measures how well people can attend to objects appearing far from fixation. The dependent measure was the score of the diagonal AW and dividing the total value by the number of the dimensions (i.e., three).

The well-established *working memory span test* (WM) by Conway et al. (2005), measures the athlete’s ability to direct attention toward the current task without getting distracted by other thoughts. More specifically, we used a counting span task (see Kane et al., 2004 for a detailed description), as the simplicity of this processing task makes it usable for almost any type of participants (Conway et al., 2005). The instructions were presented as a written text on the computer screen. The counting span task involved counting specific shapes among distractors and then remembering the count totals for later recall. Each stimulus display contained randomly arranged dark blue circles, green circles, and dark blue squares. The task of the participants was to count aloud the dark blue circles and then name the total count aloud at the end. A recall mask occurred after 2–6 stimulus displays into which participants had to fill their memorized count totals in the exact order they had been displayed in. The participants counting span score was a partial credit load score (cf. Conway et al., 2005) which represents the sum of all correctly recalled elements – whereby a correctly recalled item from a set containing two items receives 2 points, and a correctly recalled item from a set with 6 items receives 6 points – divided by the maximum possible score. The test consisted of 15 trials. The dependent measure was the score of correctly memorized objects in percentage.

The *perceptual load test* (PL) by Beck and Lavie (2005), is a measure of inhibition ability as it determines to what extent participants are distracted by stimuli which are totally irrelevant for their task. Participants performed the soccer-specific PL task (Furley et al., 2013) starting with two example blocks (one high and one low load), followed by eight experimental blocks alternating between blocks with low and high load. All participants started out with the high-load block. A fixation point of 1,000 ms was displayed before each trial located in the center of the screen immediately followed by the task display with the soccer-specific arrangement and distractor. The task displays were presented for 100 ms. Subjects were told to ignore the distractor letter and to indicate as quickly and as accurately as possible to which of the target items (the player) the dot (the ball) was allocated. The distractor always showed up on a fixation point (Beck and Lavie, 2005). Participants responded to the target stimuli either by pressing “n” or “c” on the keyboard. The subjects were instructed to press “n” for an X target and “c” for an O target. A new trial was triggered by the participant’s response or response omissions within 2 s. After each trial, feedback about incorrect responses or omissions was given by means of a computer sound. After each block, participants were reminded of the key assignments. The test consisted of 160 trials. The dependent measure was the reaction time of PL related to the condition of low and high distraction.

The *motion object tracking test* (MOT) by Alvarez and Franconeri (2007) measures up to which speed threshold participants are able to track several relevant moving objects. Participants monitored the positions of a set of moving circles on a computer monitor. The display initially contained four green circles and three blue ones (1.1° diameter). After 3 s of resting state, the blue items turned green and were identical to the targets and all circles began moving while participants tried to keep track of the positions of the initially green items. The test is adaptive so speed thresholds and number of trials depend on the players’ abilities. After 8 s the circles stopped moving and the participants had to select and mark the initially three blue circles. The dependent measure was the number of correctly tracked and marked circles. This task should reveal individual differences in the ability to divide and maintain attention on multiple independently moving objects.

#### Motor Performance Test

This diverse test battery consists of six tests (sprint 20-m, acceleration 10-m, change of direction, dribbling, ball control, and ball juggling) which assess the motor skills of soccer athletes (Höner et al., 2015).

The *Sprint test* is used to track the time an athlete needs to run 10 and 20 m as fast as possible. The test structure consists of three light barrier pairs, one pair at the start, one at the 10 m point and one at the 20 m point. The task of the athlete is to run as fast as possible through all light barriers. The dependent measure was time (in seconds) at 10 and 20 m.

The *change of direction test* is used to assess how fast the athlete is able to change directions in a preset running parkour – a fixed positioning of bars to direct the athlete in a certain change of direction. The parkour consists of a 3 m sprint to the first slalom parkour – made of three bars – then again a 3 m sprint to the second slalom parkour and then the last 3 m sprint to the finish. The time needed for this task is measured by light barriers at the starting and end point of the parkour. The dependent measure was the total time needed to absolve the parkour.

The *Dribbling test* measures the ability to dribble as fast and as accurate as possible with a ball through a preset parkour with different direction changes. The parkour and the dependent measure used for this task is the same as in the *change of direction test*.

The *Ball Control test* measures the ability to control and pass the ball in a small square as fast and as accurate as possible. The athlete is standing in the middle of the square (1.5 × 1.5 m) which consists of a bounce-wall on the left and on the right at a distance of 3 m. The task is to pass six passes alternately to the two bounce-walls as fast as possible. The passes have to be executed while standing in the middle zone and by using at least two contacts for each pass. The test is over when the last pass is received in the middle zone. The dependent measure was the total time needed to absolve the six passes.

The Ball Juggling test measures the ability to juggle the ball in a preset parkour. The parkour consists of two adjacent circles (3 × 3 m) shaped like an eight. The player starts standing in the middle of the two circles with the ball in his hand. His task is to juggle as fast as possible through the parkour. He gets a point each time he tackles the parkour without a mistake. A mistake was defined as a situation in which the ball touches the ground. The test lasts about 45 s. The dependent measure was the total number of points for successfully absolving the parkour.

We calculated one cumulated value for all cognitive tests (*Cognition Total*) and one for the motor performance tests (*Motor Total)* by adding up the scores of the dependent measures of each motor performance test and dividing the sum by the number of dependent measure variables. All values were z-standardized prior to this calculation. As reaction times for low and for high load in the PL test constitute different cognitive measures (Beck and Lavie, 2005; Furley et al., 2013), we included both values in the Cognition Total Score. *Motor Total* included the overall score of the motor test battery as well which was stated by the test leaders of the motor performance tests to gather a general impression of the performance. This is important to know, as that overall score of the motor test battery differs from the total score calculated by the authors as the first score does not include the test of acceleration.

### Statistical Analyses

Data was analyzed using IBM SPSS Statistics 23.0.0. Shapiro-Wilk test was used for testing for normal distributions. Not all variables were normally distributed, as assessed by Shapiro-Wilk’s test (*p* < 0.05). Therefore, the Spearman’s correlation coefficient test was used to investigate the correlation between the player’s cognitive and motor test results. Moreover, effect sizes (cohen’s *d*) were calculated for every correlation coefficient by transforming the *r* into a *d* value according to the formula of Ellis (2010) and values of 0.10, 0.30, and 0.50 represent small, medium and large effect size estimates (Cohen, 1998).

## Results

Descriptive statistics of each test are illustrated in Table 2.

**Table 2:** Descriptive statistics of each cognitive and motor test and their dependent measures.

	Mean value	Standard deviation
AW diagonal	4.01	3.46
MOT	988.73	232.85
PL high rt	–4.93	44.42
PL low rt	17.71	54.98
WMC	0.56	0.14
Speed (20 m)	3.43	0.17
Acceleration (10 m)	1.98	0.10
COD	7.95	0.38
Dribbling	1.44	0.95
Ball control	9.40	1.11
Ball juggling	4.77	3.77
Total score	103.97	1.91

*For all measurements, the number of participants was equal (*n* = 15), AW, attention window in cm; MOT, multiple object tracking in number of correctly tracked targets; PL, perceptual load; RT, reaction time in seconds; WM, working memory in %; COD, change of direction.*

Firstly, there was a significant correlation between the cumulated score Cognition Total and Motor Total, *r_s_* (13) = 0.614, p = 0.015. Therefore, superior performance in the cognition tests significantly correlates positively with superior performance in the motor tests. Due to this result we further checked for correlations that cause this finding.

In terms of diagonal AW there were no statistically significant correlations between the motor tests except for the dribbling test [*r_s_* (13) = 0.656, *p* = 0.008] as depicted in Table 3. The MOT task was not significantly associated with any of the other tests. Furthermore, there were no significant correlations between the PL reaction times and all other tests. However, there were significant correlations between the WMC test and the test of Dribbling [*r_s_* (13) = 0.562, *p* = 0.029], Ball control [*r_s_* (13) = 0.669, *p* = 0.006], Ball juggling [*r_s_* (13) = 0.727, *p* = 0.002], and the Total Score [*r_s_* (13) = 0.553, *p* = 0.033]. Thus, superior performance in the WMC test significantly correlates positively with superior performance in the motor tests.

**Table 3:** Correlations *r*_s_ between cognitive and motor tests.

	Speed (20 m)	Acceleration (10 m)	COD	Dribbling	Ball control	Ball-Juggling	Total score
MAW diagonal							
Correlation coefficient	0.087	–0.014	0.339	0.656**	0.380	0.098	0.395
Sig. (twofold)	0.758	0.961	0.216	0.008	0.162	0.729	0.145
Effect size (d)	0.175	–0.027	0.721	1.74	0.822	0.197	0.860
MOT							
Correlation coefficient	–0.047	–0.126	–0.032	0.125	0.146	0.123	0.175
Sig. (twofold)	0.869	0.656	0.909	0.657	0.603	0.664	0.533
Effect size (d)	–0.093	–0.254	–0.064	–252	–294	–248	0.356
PL high reaction time							
Correlation coefficient	–0.029	–0.143	0.211	0.318	0.425	0.418	0.396
Sig. (twofold)	0.919	0.610	0.449	0.248	0.114	0.121	0.143
Effect size (d)	–0.058	–0.289	0.432	0.671	0.939	0.921	0.863
PL low reaction time							
Correlation coefficient	–0.095	–0.056	–0.409	–0.396	0.075	0.168	0.021
Sig. (twofold)	0.737	0.844	0.130	0.143	0.791	0.551	0.940
Effect size (d)	–0.191	–0.112	–0.895	–0.863	0.150	0.341	0.430
WMC							
Correlation coefficient	–0.260	–0.249	0.197	0.562*	0.669**	0.727**	0.553*
Sig. (twofold)	0.350	0.371	0.480	0.029	0.006	0.002	0.033
Effect size (d)	–0.539	–0.513	0.402	1.39	1.81	2.12	1.33

	**Motor total**						

Cognition Total
Correlation coefficient	0.614*						
Sig. (twofold)	0.015						
Effect size (d)	1.56						

*^∗∗^The correlation is significant at 0.01 level (twofold). ^∗^The correlation is significant at 0.05 level (twofold). For all measurements, the number of participants was equal (*n* = 15). COD, change of direction; AW, attention window; MOT, multiple object tracking; PL, perceptual load; WMC, working memory capacity.*

## Discussion

The current study addressed the relationship between cognitive functioning and specific motor abilities in elite youth soccer players. The aim was to expand the knowledge of the relationship between basic cognitive skills and soccer specific motor skills. Results showed that the diagonal AW was positively correlated with dribbling performance. This may suggest that athletes who have a wider AW also have advanced dribbling skills. Moreover, these findings could imply that a broad AW enhances the players’ skills regarding highly demanding motor tasks, because they may be able to perceive many optical stimuli in their visual AW. This may enable them to execute early reactions in their sensorimotor system to make their performance more efficient. For example, in a game situation where the athlete is dribbling and simultaneously has to keep an eye on the ball, his teammates and his opponents. In this case a broad AW could be beneficial for example to avoid contact with opponents and dribbling in spaces already covered by teammates. These results are in line with previous meta-analysis (Voss et al., 2010; Scharfen and Memmert, 2019) which implicated superior cognitive abilities in elite athletes. Another positive relationship was reported for WMC and dribbling as well as for ball control, ball juggling, and total score. Especially these findings regarding WMC are in line with studies examining cognitive functions in elite athletes mentioned earlier. Previous research for example indicated (a) that a higher WM capacity is associated with a superior athletic performance as well as (b) that time spent in organized sports positively correlates with WM (e.g., Verburgh et al., 2014, 2016). Nevertheless, there are also other studies which did not indicate this relationship (e.g., Furley and Memmert, 2010; Balakova et al., 2015). Additionally, the missing correlation of the motor tests with the MOT and the PL test could be due to the fact that the motor tasks do not include similar demands. For example, in the motor performance tests used here there is no task in which multiple objects or players need to be tracked simultaneously. Therefore, there is no situation that requires similar skills or has the same task structure like the MOT. Although several studies related to the perception-action coupling approach (Renshaw and Davids, 2004; Pinder et al., 2009; Davids et al., 2013) already proved the link of specific perceptual abilities and performance, this is the first study to our knowledge that shows a positive correlation between the cumulated scores of all basic cognitive and all motor tests which could point at a strong interplay of physical and psychological skills. These results are in line with mutually influencing cognition- and motor-networks on a basic functional (Leisman et al., 2016; Ptak et al., 2017) and structural level (Koziol et al., 2012; Bigelow and Agrawal, 2015; Gao et al., 2018). Furthermore, they could also be a hint for the use of similar neural networks (Hanakawa, 2011) and of the same brain regions (Leisman et al., 2016) when carrying out different cognitive tasks and motor skills. Additionally, these findings are in agreement with Grooms and Onate (2016) who state the ability to maintain motor control in the unpredictable sport environment demands complex central nervous system integration of a constantly changing profile of sensory inputs. Moreover, it is also in consonance with their statement that the incorporation of cognitive elements ranging from dual tasks, responding to stimuli, anticipation, decision making, and programming motion relative to external targets may degrade neuromuscular control relative to movement without such factors. In terms of developmental motor-cognition interactions these findings point at the same direction as previous research did with (1) younger children (Jansen and Heil, 2010; Lehmann et al., 2014; Gottwald et al., 2016), (2) cognitive improvements as a function of physical exercise (Hillman et al., 2009; Tomporowski et al., 2015), and (3) strong motor-cognition relations in pre-pubertal children (i.e., under 13 years of age) (for review see van der Fels et al., 2015).

Although the results are based on a cross-sectional study and await replication in a design that allows causal interpretations, the data unveils a possible explanation for differences in performance among elite youth soccer players in terms of their cognitive function and their specific motor skills. WMC and AW may prove relevant for talent identification purposes as they are strongly associated with ball juggling, ball dribbling, and especially the total motor skill score and pace, which are all of major interest in professional soccer clubs. By adding these cognitive tests to the physical ones (those who correlated significantly, i.e., dribbling, ball control, ball juggling, and the total score) the impressions and values derived from the physical tests could be strengthened and, besides, the information about the players’ profiles in terms of cognitive function would be extended. In terms of talent development, playing soccer at a high level of performance each day, that is in a talent selection team of a professional soccer club, seems to be associated with the development of most of the cognitive skills. This could indicate that these cognitive skills may be crucial for talent development and could be promoted via these talent programs of professional soccer clubs – a positive reciprocal development. Nevertheless, we cannot draw causal conclusions based on our data as talent development might be influenced by a third variable. However, to the best of our knowledge, this study is the first that examines the combination of several cognitive functions and soccer-specific motor skills in young soccer experts. Future studies are clearly needed to investigate this promising relationship further.

We should also acknowledge limitations of the present study. The used motor test analyzes basic soccer-specific motor skills like dribbling and juggling which are able to distinguish elite from recreational soccer players, but not elite from sub-elite (Meylan et al., 2010). The study, thus, does not cover the whole spectrum of the complex soccer game. One example is that no HEFs were assessed which are crucial for the complex game as well (Vestberg et al., 2017). Therefore, some core tactical abilities, such as MOT in a dynamic surrounding like small-sided games are missing. Furthermore, although the change of direction test is well validated, it is a limitation, as it lacks external stimuli on which the changes of direction depend in a real game situation. The differentiation between change of direction and agility is crucial in this regard (Haff and Tripplet, 2016). There is no situation in the game, in which a player has to change his direction in a preset order. Additionally, a high number of correlations in our study were not significant. Thus, more replication research in this field is clearly needed (Klein et al., 2012). Furthermore, the study lacks highly statistical power as the unique sample is relatively small due to the fact that elite youth soccer players have been examined whose accessibility is strictly limited for most of the time.

Consequently, there are several recommendations for future research derived from limitations of this study. First, linking cognitive test results (especially HEF) to (1) that are able to measure more complex and diverse soccer- and sport-specific skills. This is necessary to expand the knowledge about these correlations (e.g., small-sided games and agility test with external stimuli) and (2), objective performance measurements (e.g., assessment of performance during game play), and to strengthen possible relationships and replicate findings from previous studies (Vestberg et al., 2012, 2017). However, it should be noted that performance objectives like scored goals are difficult to measure in young athletes due to highly varying positions of players and due to the fact that the likelihood of scoring goals varies highly depending on the position. Regarding (1), it will be a challenge to include those tests as there may occur problems in terms of objectivity and reliability. Secondly, longitudinal measurements with a larger number of players are necessary to examine the age-related interaction of cognitive functions, soccer-specific motor skills, and their development. Especially considering the individual timing of maturation of cognitive functions (Best et al., 2009). Moreover, using longitudinal study designs would enable researchers to search for additional influential factors as well as to conclude and uncover for further causal relationships as this possibility is very limited in cross-sectional studies. Finally, investigations of HEFs are needed as well as they are an important aspect of the complex game as well (Vestberg et al., 2017).

To summarize, we found that the cognitive functions AW and working memory are partly associated with some specific and core motor skills, whereas the sum of all cognitive, and all motor skills are strongly correlated as well. Additionally, the cognitive test, MOT and PL test, did not show any relation to the tested motor skills. Although one has to keep in mind that this only a first attempt to understand the relationship between cognitive and motor behavior one may have a look at the direction at which these results could point. Namely, these findings could be important from both a theoretical and a practical perspective. From a theoretical point of view, this may highlight the importance of cognitive training models that are based on neuro-cognitive knowledge as well as the need for more sophisticated models and theories that explain the relationship of movements i.e., technique and cognition. The usage of such training models increases the cognitive functions and possibly the motor technique skills, which is in line with recommendations of van der Fels et al. (2015) and with the results above showing correlations of both abilities. The arising need for these cognitive training models is in line with the increasing evidence that some of these cognitive functions like AW and MOT (Romeas et al., 2016; Savelsbergh, 2017) as well as working memory (Klingberg, 2010) are trainable. Nevertheless, the transfer to the real game is not clearly established for every improvement yet. Furthermore, research suggests that a combination of cognitive and physical training is more beneficial for the athletes in terms of cognitive functions, mental health and neurogenesis than only one of them conducted separately (Curlik and Shor, 2012).

From a practical point of view, knowledge about the relationship between cognition and motor skills, in other words between the brain-muscle interplay, could possibly help sport clubs to be able to scout for talents, and new players in a more effective and holistic way. This sophisticated scouting system could be created by adding a cognitive scouting or test tool to the categories of technique, athletics, and tactics. Adding this cognitive tool is backed up by studies that report a high linkage and overlap between cognitive functions and game intelligence, which is crucial for success in elite sports, and still hardly measurable (e.g., Vestberg et al., 2012, 2017). Additionally, this knowledge may be used by coaches to enhance their players’ cognitive abilities and eventually some of their motor skills, as well as improving working memory for general motor skills. Referring to this, individual soccer training programs could be created based on these relationships to enhance soccer performance on the pitch.

Furthermore, as these results may point in the same direction as the perception-action coupling approach, it could perhaps help coaches to create training programs and exercises which do not isolate sport specific perception (i.e., cognitive functions) and action (i.e., motor skills) but rather enhance both in unison as these couplings are required and highly challenged in real game situations. Moreover, training those couplings and incorporating cognitive elements may not only enhance performance (Belling and Ward, 2015; Broadbent et al., 2015; Grooms et al., 2015; Appelbaum and Erickson, 2018; Hadlow et al., 2018) but also prevent athletes from injuries (Grooms et al., 2015; Grooms and Onate, 2016).

Further research should provide more evidence for elite youth athletes as specifically these early years in a player’s career are crucial for the development of the athlete’s cognitive abilities. The sensitive learning phases occur during this period of time which highlights the importance of this age group for further development of the athlete’s skills.

## Ethics Statement

This study was carried out in accordance with the recommendations of the ‘ethics committee of the German Sport University Cologne’ with written informed consent from all subjects. All subjects gave written informed consent in accordance with the Declaration of Helsinki. The protocol was approved by the ‘ethics committee of the German Sport University Cologne’.

## Author Contributions

All authors listed have made a substantial, direct and intellectual contribution to the work, and approved it for publication.

## Conflict of Interest Statement

The authors declare that the research was conducted in the absence of any commercial or financial relationships that could be construed as a potential conflict of interest.
